# Rehabilitation after lumbar spine surgery in adults: a systematic review with meta-analysis

**DOI:** 10.1186/s40945-023-00175-4

**Published:** 2023-10-16

**Authors:** Tiziana Manni, Nicola Ferri, Carla Vanti, Silvano Ferrari, Ilaria Cuoghi, Claudia Gaeta, Isabella Sgaravatti, Paolo Pillastrini

**Affiliations:** Department of Biomedical and Neuromotor Sciences (DIBINEM), Alma Mater Studiorum, University of Bologna, 40138 Bologna, Italy

**Keywords:** Rehabilitation, Exercise therapy, Low back pain surgery, Post-operative period, Disc herniation, Spondylolisthesis, Stenosis, Meta-analysis

## Abstract

**Background:**

The role of rehabilitation after surgery in patients with low back pain is well recognized. The aim of this systematic review is to summarize and update the existing evidence according to the type of clinical condition and rehabilitation approach.

**Methods:**

This systematic review included RCTs on the effectiveness of rehabilitation after surgery for lumbar disc herniation, spinal stenosis, and spondylolisthesis. We searched the literature for randomized controlled trials indexed in MEDLINE, Embase, CINHAL, CENTRAL, Scopus, PEDro, and Web of Science databases, up to April 15, 2023. We used Cochrane Risk of Bias 2.0 tool to assess each study. We conducted a quantitative synthesis when population, intervention, control, and outcome were sufficiently homogeneous; otherwise, we conducted a qualitative analysis.

**Results:**

Forty-five studies (3.036 subjects) were included and analyzed according to the population considered: lumbar stenosis (1 trial), spondylolisthesis (3 trials), and disc herniation (41 trials). Regarding lumbar stenosis, a supervised active exercise program appears to improve outcomes related to pain, disability, and quality of life both in the short- and mid-term (1 study, *n* = 60). Concerning spondylolisthesis, kinesiophobia is reduced in the home exercises group compared to usual care, at 3-months follow-up (3 studies, *n* = 98). For disk herniation, supervised exercises are better than non-supervised exercises to reduce pain (MD -1.14; 95% CIs -1.65, -0.62; 5 trials, *n* = 250) and disability (SMD -0.70; 95% CIs -1.14, -0.26; 4 trials, *n* = 175). Supervised exercises are better than advice in reducing pain (SMD -0.91; 95% CIs -1.61, -0.21; 5 trials, *n* = 341) and disability (SMD -0.80; 95% CIs -1.59, -0.01; 4 trials, *n* = 261), in the short-term. Supervised exercises are equal to no treatment in reducing pain and disability, at 3 and 6 months after intervention (2 trials, *n* = 166). These results are supported by a very low to low quality of evidence.

**Conclusions:**

Our research suggests that supervised exercise may be effective in improving patient’s pain and disability after lumbar surgery, but RCTs regarding lumbar spinal stenosis and lumbar spondylolisthesis are still scarce, with significant heterogeneity of proposed interventions.

**Supplementary Information:**

The online version contains supplementary material available at 10.1186/s40945-023-00175-4.


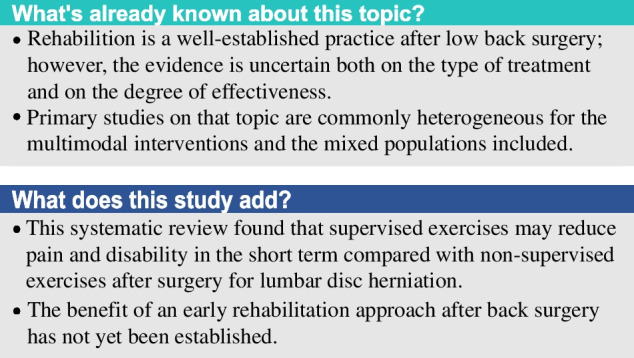


## Background

Pain and disability caused by lumbar disc herniation, spondylolisthesis, and stenosis are the most frequent reasons for lumbar surgery referral [1]. Nevertheless, surgical treatment itself may provoke muscle atrophy and weakness [2], stiffness [3], fear of movement, and consequent disability [4] in activities of daily living [5]. Therefore, postoperative physical and mental health may be affected over than expected [6]. For this reason, different rehabilitation programs have been proposed, with the aim to accelerate the resolution of symptoms, especially pain, promoting functional recovery and return to work, reassuring patients, and finally preventing chronic pain, complications, and relapses [7].

The effectiveness of rehabilitation after lumbar surgery has been investigated by several studies. A Cochrane review on lumbar disc herniation surgery [8] found no differences between supervised and home exercises for pain, disability, or perceived overall effect. Moreover, none of the included studies reported an increase in the reoperation rate after the first lumbar surgery.

Another systematic review by Santana-Ríos and colleagues [9] concluded that an early rehabilitation program is recommended for patients undergoing first microdiscectomy, due to the excellent results and the absence of adverse effects. More specifically, positive reinforcement / cognitive intervention along with exercise was considered an effective treatment, potentially an alternative to spinal fusion in patients with symptom recurrence, after the first surgery.

Another Cochrane review on the effectiveness of rehabilitation after lumbar spinal stenosis surgery [10] suggested that active rehabilitation is more effective than usual care in improving back-related functional status, in the short- and long-term. Similar results emerged for secondary outcomes, including short-term improvement in low back pain (LBP) and long-term improvement in both LBP and leg pain, although limited impact was observed for the overall health improvement. The clinical relevance of these effects was reported as medium to small. These conclusions were limited by the few number (# 3) of relevant studies identified. More recently, the Danish Guidelines published in 2019 [11] recommended the use of supervised exercise after surgery for lumbar stenosis, due to its effectiveness on overall health, although no evidence of effects on neurogenic pain was found. These Guidelines did not recommend manual therapy.

With regard to the effectiveness of rehabilitation after surgery for lumbar spondylolisthesis, the North American Spine Society (NASS) Evidence-based Clinical Guidelines [12] concluded that post-surgical rehabilitation (including physical exercise, spinal mobilization/ manipulation, or psychosocial interventions) in degenerative lumbar spondylolisthesis is unresponsive due to the paucity of evidence. These guidelines stated that no systematic review concerning rehabilitation after surgery for isthmic spondylolisthesis is available at present.

All the cited reviews and Guidelines pointed out the need for further, high-quality randomized controlled trials (RCTs), to make their conclusions stronger.

The objective of this review is to estimate the effectiveness of various post-surgical rehabilitation interventions on pain, function, disability, and health in adults after a first surgery for lumbar disc herniation, lumbar spinal stenosis, or lumbar spondylolisthesis. In addition, we aimed to collect the occurrence and severity of adverse events or complications related to post-surgical rehabilitation.

## Methods

This systematic review protocol was registered on the PROSPERO database (code ___________CRD42021278556).

### Inclusion criteria

#### Types of studies

We included only RCTs.

#### Types of participants

We included studies on adults (e.g. people older than 18 years) who underwent first lumbar surgery for lumbar disc herniation, lumbar spinal stenosis, or lumbar spondylolisthesis.

#### Setting

We considered all settings in which post-surgical rehabilitation can be carried out, such as Hospital Departments of Physical and Rehabilitation Medicine, Physiotherapy and Rehabilitation Outpatient Clinics, Physiotherapy and Rehabilitation Professional Practices, both public and private, as well as home settings in case of self-treatment.

#### Publication date

We considered all articles published from inception to the search date.

#### Language

We included all articles without language limitations. Collaborating translators, in case of needs, offered assistance.

#### State of publication

We included all published RCTs.

#### Types of interventions

According to the World Health Organization (WHO) [13], rehabilitation is defined as “a set of interventions aimed at optimizing functioning and reducing disability in subjects with health conditions interacting with their environment”. Rehabilitation includes passive/manual techniques and active exercise, with or without the use of physical/mechanical agents, splinting or bracing, education, and strategies for improving self-efficacy. Categorization of rehabilitation interventions is neither simple nor exhaustive, so the authors included in the search strategy all the terms that could includeprocedures used by this discipline.

#### Comparison

Comparisons can be among different physiotherapy or pharmacological interventions, wait-and-see strategies, placebo, sham, or no intervention.

#### Types of outcome measures

Primary outcomes were pain measured through the Visual Analogue Scale (VAS) or the Numeric Rating Scale (NRS), and physical function (e.g. the range of motion, measured with a manual or digital goniometer). Secondary outcomes were disability, measured with the Roland and Morris Disability Questionnaire or the Oswestry Disability Index (ODI), and the perceived health status measured through the Short Form-36 (SF-36), or the EuroQol*-*5 Dimension (EQ-5D).

In addition, we collected adverse events, defined as any adverse symptoms, signs, or temporary illness associated with treatment. Indirect harms were also considered, where an intervention delayed a diagnosis or treatment, and that delay represented potential harm. The need for additional surgery was also considered.

### Search strategy for identification of studies

We searched MEDLINE, Embase, CINHAL, CENTRAL, Scopus, PEDro, and Web of Science databases. Different search strategies were used for each database; these included a combination of specific terms (e.g., MeSH in MEDLINE) and free text words to capture key concepts in titles and abstracts (Additional file 1).

We uploaded records into Endnote Web Reference Management software (Clarivate Analytics, Philadelphia, Pennsylvania, USA) to manage articles and remove duplicates. Two independent reviewers screened the articles based by titles and abstracts using RAYYAN.AI website tool, then by full texts according to inclusion and exclusion criteria. Any disagreements were resolved by a third reviewer. We completed this process by also looking through the reference lists of included studies. Review members met before starting the screening process to ensure agreement on the application of pre-defined inclusion criteria. In addition, bimonthly meetings were held to discuss the screening process and any unforeseen challenge.

### Data extraction

Two independent reviewers extracted the following data from all the included studies:

• populations: socio-demographic information (e.g. age, sex, education, occupation, culture), health status (e.g. comorbidities, previous surgery), geographic region, type of pre-surgical pathology (e.g. lumbar disc herniation, spondylolisthesis, or spinal stenosis), surgical procedure (e.g. microdiscectomy, decompressive laminectomy/laminotomy/foraminotomy, spinal fusion);
• interventions: type (e.g. endurance exercise, manual therapy, physical agents), context (e.g. hospital, outpatient clinic), start time (weeks or months after surgery), type of clinician providing the surgery, duration (weeks or months), dosage (times per week);
• comparisons: wait and see, placebo, no treatment, other rehabilitation treatments, drugs;
• outcomes of interest;
• adverse events and further surgery;
• key findings stratified by patient characteristics, pre-surgical pathology, type of surgery, and specific type of post-surgical rehabilitation or care programs, where possible.

In case there is more than one outcome measure to evaluate the same construct, different analyses were performed. Any disagreements between the reviewers were resolved through discussion or with the help of a third expert reviewer. Studies’ authors were contacted if there were missing data.

### Data synthesis

Reviewers considered pooling studies in a meta-analysis when there was a clinical and methodological homogeneity with respect to the PICO models.

We performed statistical analyses with the REVMAN 5.4 software (produced by the Cochrane Collaboration), using post-treatment data at different endpoints; we categorized short-term (up to 6 months) and long-term (at least 1 year). Endpoints for all studies are given in Additional file 2.

When outcomes were continuous, we calculated the mean difference (MD) if the outcome in all studies was measured with the same scale; otherwise, a standardized mean difference (SMD) was used. We set a 95% confidence interval.

When the PICOs of the included trials implied a common effect between the studies, a fixed effect was chosen; otherwise, a random effect analysis was performed.

In two studies [14, 15] only change-from-baseline scores were reported and we pooled them with other trials’ post-treatment data, backed up by sensitivity analysis (Cochrane, 7.7.3.1; da Costa, 2013).

In case of missing data, first, the reviewers contacted the authors to obtain the missing information; in the absence of any response, the data were extracted when presented only in the form of graphs by using the Web Plot Digitizer ver. 4.5 software (produced by WebPlotDigitizer, 2020). A study [16] reported only median and interquartile range; the mean and standard deviation were imputed considering the sample size as recommended by Hozo et al. [17].

Statistical heterogeneity was calculated by the I^2^ test and visual analysis of confidence intervals’ overlap. When the reviewers found a moderate to substantial level of heterogeneity (I^2^ > 50%), subgroup and sensitivity analyses were performed to investigate the source of heterogeneity.

When clinical heterogeneity was relevant, we did not perform meta-analysis instead, we did a qualitative synthesis.

### Assessment of risk of bias and quality of evidence

To assess the risk of bias we used the RoB 2.0 Cochrane tool, which contains five domains (Randomization process, Deviations from the intended interventions, Missing outcome data, Measurement of the outcome, Selection of the reported result). If the details reported in a study were insufficient, the reviewers contacted the authors of the original study to obtain more information before conducting the risk of bias assessment. This process was carried out by two independent reviewers; if there was disagreement, a third reviewer intervened to discuss and resolve the debate. At the end of this process, a summary table was created.

The reviewers judged the quality of evidence for all findings through the GRADE (Grading of Recommendations, Assessment, Development and Evaluations) tool and created SoF (Summary of Findings) tables accordingly.

## Results

**Fig. 1:**
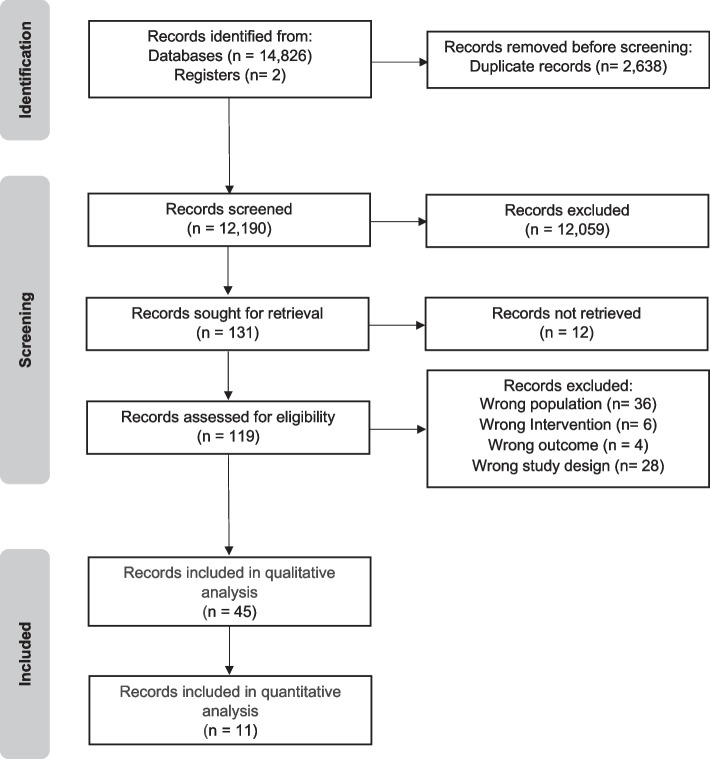
Flow chart of studies selection

The search was performed from June to October 2021 and updated on April 15, 2023. 14,826 studies were identified and 2 more studies were added by checking the references of the included studies. After removal of duplicates and screening process, 45 studies were included and grouped according to the population considered: lumbar stenosis (1 article), spondylolisthesis (3 articles), and disc herniation (41 articles). Of the latter 41 articles, 11 studies were considered for quantitative analysis, while all other studies were analyzed only qualitatively, due to the high heterogeneity of the intervention, comparison, and outcomes (see Fig. 1).

### Description of studies and results

A complete description of the included studies is available in Additional file 2, while the risk of bias assessment is presented in Table 1 and GRADE assessment in Tables 2, 3 and 4.

**Table 1:**
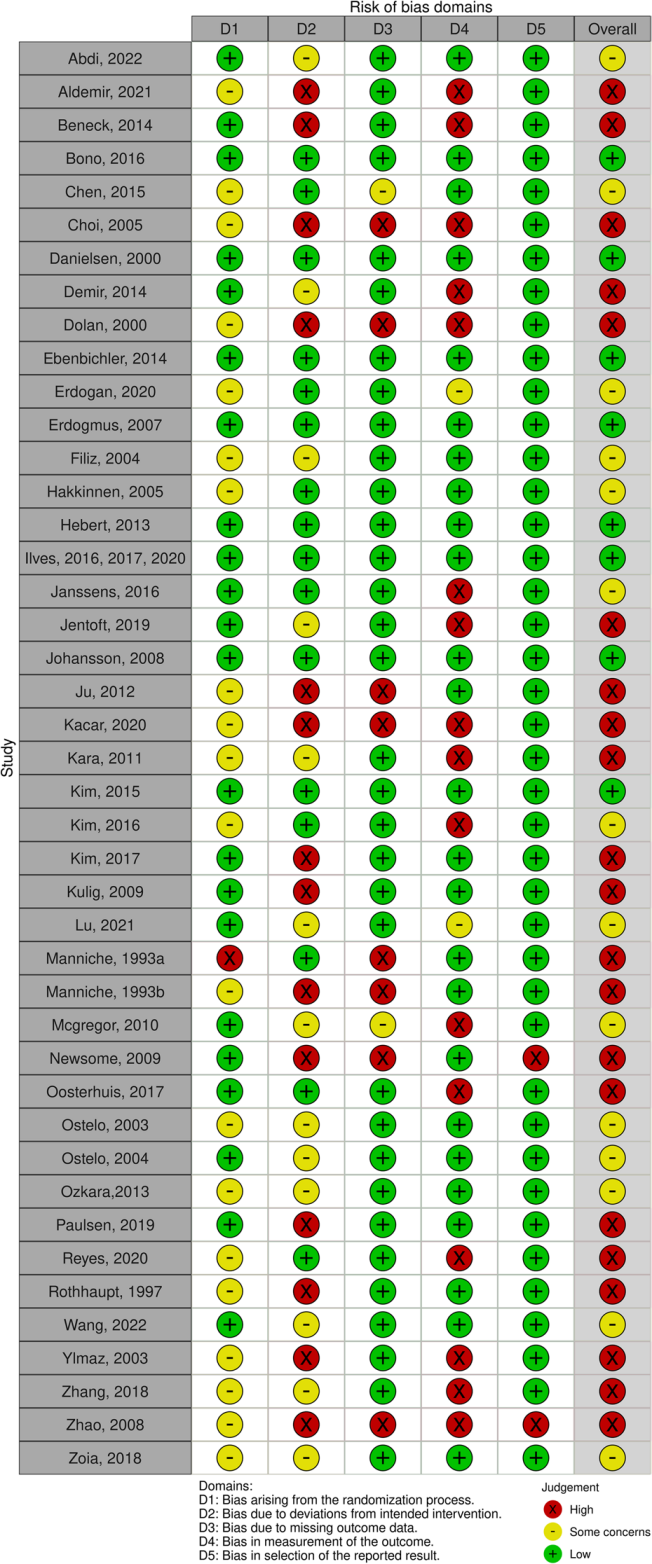
Risk of bias of the included studies

**Table 2:** GRADE assessment: supervised exercise compared to non-supervised exercise

Supervised exercise compared to non-supervised exercise for patients after surgery for lumbar disk herniation Patient or population: patients after surgery for lumbar disk herniation Setting: Hospital Intervention: supervised exercise Comparison: non-supervised exercise					
Outcomes	№ of participants studies) Follow-up	Certainty of the evidence (GRADE)	Relative effect (95% CI)	Anticipated absolute effects	
				Risk with nonsupervised exercise	Risk difference with supervised exercise
Pain assessed with: Visual Analogue Scale (cm) Scale from: 0 to 10 follow-up: mean 4 months	250 (5 RCTs)	⨁⨁◯◯ Low^a,b^	-		MD 1.14 lower (1.65 lower to 0.62 lower)
Disability follow-up: mean 4 months	175 (4 RCTs)	⨁⨁◯◯ Low^b,c^	-	-	SMD 0.7 SD Lower (1.14 lower to 0.26 lower)
Lumbar Mobility assessed with: Schober Test (cm) follow-up: mean 3 months	68 (2 RCTs)	⨁⨁◯◯ Low^b,d^	-		MD 0.27 lower (0.7 lower to 0.16 higher)

*CI* confidence interval, *MD* mean difference, *SMD* standardised mean difference

GRADE Working Group grades of evidence

High certainty: we are very confident that the true effect lies close to that of the estimate of the effect.

Moderate certainty: we are moderately confident in the effect estimate: the true effect is likely to be close to the estimate of the effect, but there is a possibility that it is substantially different.

Low certainty: our confidence in the effect estimate is limited: the true effect may be substantially different from the estimate of the effect.

Very low certainty: we have very little confidence in the effect estimate: the true effect is likely to be substantially different from the estimate of effect.

^*^The risk in the intervention group (and its 95% confidence interval) is based on the assumed risk in the comparison group and the relative effect of the intervention (and its 95% CI).

Explanations

^a^3 of 5 studies are at high risk of bias

^b^N<400

^c^2 of 4 studies at high risk of bias

^d^2 of 3 studies at high risk of bias

**Table 3:** GRADE assessment: supervised exercise compared to advice

Supervised exercise compared to advice for patients after surgery for lumbar disk herniation Patient or population: patients after surgery for lumbar disk herniation Setting: Hospital Intervention: supervised exercise Comparison: advice					
Outcomes	№ of participants studies) Follow-up	Certainty of the evidence (GRADE)	Relative effect (95% CI)	Anticipated absolute effects	
				Risk with advised	Risk difference with Supervised exercise
Pain follow up: mean 4 months	341 (5 RCTs)	⨁⨁◯◯◯ Very low^a,b,c^	-	-	SMD 0.91 SD lower (1.61 lower to 0.21 lower)
Disability follow-up: mean 4 months	261 (4 RCTs)	⨁◯◯◯ Very low^b,c,d^	-	-	SMD 0.8 SD lower (1.59 lower to 0.01 lower)

*CI* confidence interval, *MD* mean difference, *SMD* standardised mean difference

GRADE Working Group grades of evidence

High certainty: we are very confident that the true effect lies close to that of the estimate of the effect.

Moderate certainty: we are moderately confident in the effect estimate: the true effect is likely to be close to the estimate of the effect, but there is a possibility that it is substantially different.

Low certainty: our confidence in the effect estimate is limited: the true effect may be substantially different from the estimate of the effect.

Very low certainty: we have very little confidence in the effect estimate: the true effect is likely to be substantially different from the estimate of effect.

^*^The risk in the intervention group (and its 95% confidence interval) is based on the assumed risk in the comparison group and the relative effect of the intervention (and its 95% CI).

Explanations

^a^2 studies at high risk and 2 studies with some concerns

^b^high heterogeneity

^c^n<400

^d^2 studies at high risk, 1 study with some concerns

**Table 4:** GRADE assessment: supervised exercise compared to no treatment

Supervised exercise compared to advice for patients after surgery for lumbar disk herniation Patient or population: patients after surgery for lumbar disk herniation Setting: Intervention: supervised exercise Comparison: no treatment					
Outcomes	№ of participants studies) Follow-up	Certainty of the evidence (GRADE)	Relative effect (95% CI)	Anticipated absolute effects	
				Risk with nonsupervised exercise	Risk difference with supervised exercise
Pain assessed with: Visual Analogue Scale Scale from: 0 to 10 follow-up: mean 3 months	166 (2 RCTs)	⨁◯◯◯ Very low^a,b^	-		MD 0.34 lower (7.32 lower to 6.63 higher)
Disability follow-up: mean 3 months	166 (2 RCTs)	⨁◯◯◯ Very low^a,b^	-	-	SMD 0.11 SD lower (0.42 lower to 0.19 higher)
Pain assessed with: Visual Analogue Scale Scale from: 0 to 10 follow-up: mean 6 months	166 (2 RCTs)	⨁◯◯◯ Very low^a,b^	-		MD 9.28 higher (2.78 higher to 15.77 higher)
Disability follow-up: mean 6 months	166 (2 RCTs)	⨁◯◯◯ Very low^a,b,c^	-	-	SMD 0.06 SD lower (0.71 lower to 0.59 higher)

*CI* confidence interval, *MD* mean difference, *SMD* standardised mean difference

GRADE Working Group grades of evidence

High certainty:we are very confident that the true effect lies close to that of the estimate of the effect.

Moderate certainty:we are moderately confident in the effect estimate: the true effect is likely to be close to the estimate of the effect, but there is a possibility that it is substantially different.

Low certainty:our confidence in the effect estimate is limited: the true effect may be substantially different from the estimate of the effect.

Very low certainty:we have very little confidence in the effect estimate: the true effect is likely to be substantially different from the estimate of effect.

^*^The risk in the intervention group (and its 95% confidence interval) is based on the assumed risk in the comparison group and the relative effect of the intervention (and its 95% CI).

Explanations

^a^all studies at high risk of bias

^b^n<<400

^c^moderate heterogeneity, discordant point estimates

#### Lumbar spinal stenosis

##### Comparison: education plus exercises vs usual care

One RCT on 60 participants was included in this review [18]. Patients were randomly allocated in a peri-operative group and a control group. The peri-operative group received preoperatively and early postoperative education, postural awareness, mobilization strategies, core stability exercises, muscle strengthening exercises, deep breathing exercises, and exercises oriented to the activities of daily living, for 30 min a day during hospitalization. The control group only received instructions on post-operative care (usual care protocol).

Compared with the control group, the peri-operative group demonstrated a significant decrease in pain and higher quality of life (mental health), at 1-month follow-up. On the other hand, the control group demonstrated better scores for both domains of quality of life from 1-month follow-up onwards. The average level of disability improved significantly in both groups at 6-month follow-up. All patients changed their functional capacity. The main improvement was demonstrated in the five repeated sit-to-stands exercises, with no difference between the peri-operative and control group. This study is considered to have a moderate risk of bias, due to the randomization process (unclear) and missing data in the results.

#### Lumbar spondylolisthesis

##### Comparison: home exercises versus usual care

The three articles included on rehabilitation after spondylolisthesis surgery [19–21] were considered as one study, because they were conducted on the same sample, but on different outcomes. This sample included 98 patients with isthmic (31 patients) or degenerative (67 patients) spondylolisthesis. The aim of the study was to investigate the effectiveness of postoperative 12-month home exercises compared with usual care on pain, disability, quality of life, and kinesiophobia

During the three months after surgery, before the start of physiotherapy intervention, low back and leg pain intensity decreased in both samples. During the 12 months of physiotherapy intervention, no changes in low back or leg pain were observed within the groups. Disability, measured with the ODI scale, decreased in both groups; no differences in quality of life were found at any time point between groups. At 3-month follow-up there was a reduction in kinesiophobia in the home exercise group compared with the usual care group. This study was assessed as affected by a low risk of bias.

#### Lumbar disc herniation

Forty-one RCTs were included in this systematic review [14–16, 22–60], with a total of 3036 participants. Twenty-five studies evaluated the effectiveness of rehabilitation in an early phase after surgery, sixteen RCTs analyzed the interventions within one year and only one RCT studied physiotherapy delivered one year after surgery. The rehabilitation programs were heterogeneous; most studies focused on comparing supervised exercise programs with home-based exercise programs. In addition, education programs were investigated, with a particular attention on the effectiveness of cognitive behavioral therapy.

#### Quantitative analysis

We pooled 13 trials for three different comparisons: supervised exercises vs non-supervised exercises (6 trials), supervised exercises vs advice (5 trials), and supervised exercises vs no treatment (2 trials). Data included in the meta-analyses were collected at 3 and 6 months after surgery.

##### Comparison: supervised vs non-supervised exercises

Six trials reported results on short-term effects of supervised exercises vs non-supervised exercises; in one trial [18] the treatment started early after surgery and it was therefore clinically heterogeneous. We pooled the effect from the five remaining trials [14, 22–25] with a similar delayed approach (mean ± SD = 4.4 ± 0.8 weeks post-surgery).

**Fig. 2:**
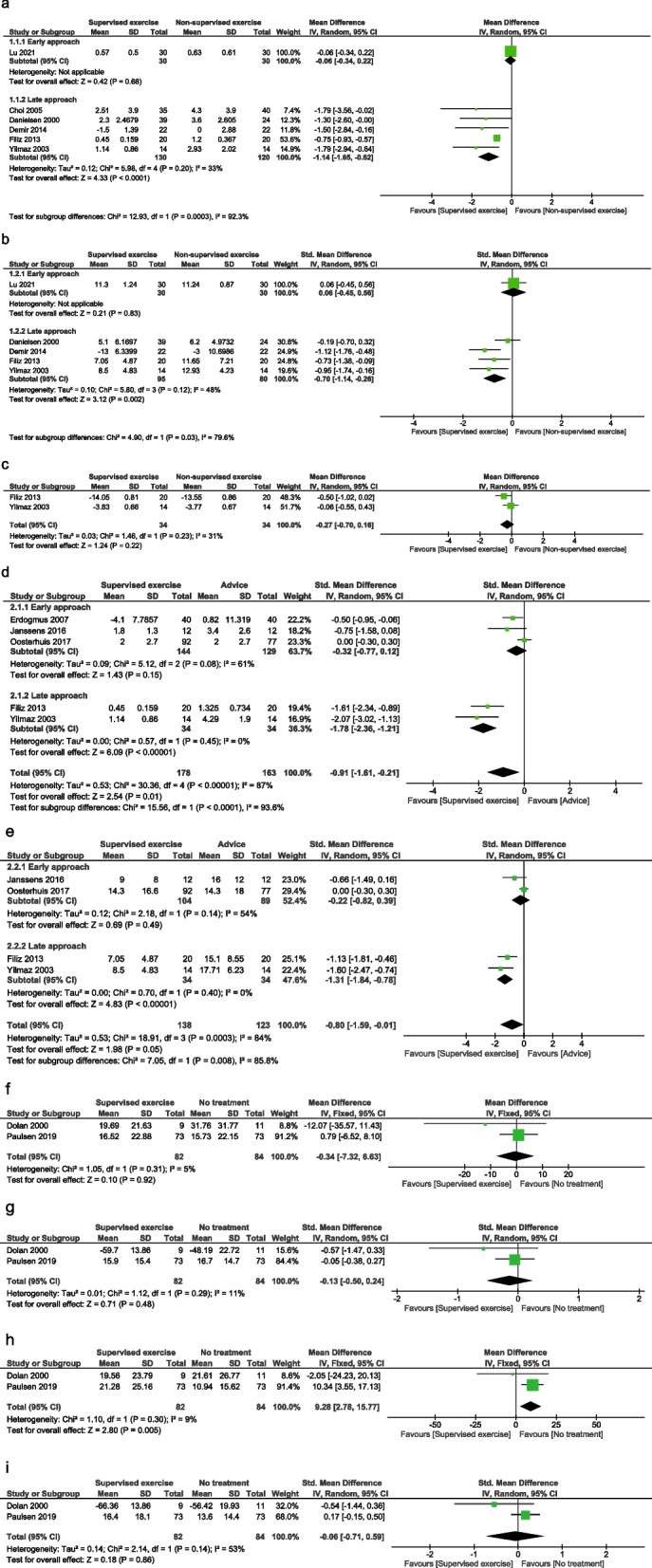
Results of meta-analyses

There is low quality evidence that supervised exercises reduce pain in the short-term (MD -1.14; 95% CIs -1.65, -0.62; I^2^ = 33%; 5 trials, *n* = 250) (Fig. 2a). This finding is statistically significant although the clinical impact may be minimal.

There is low quality evidence that supervised exercises reduce disability in the short-term (SMD -0.70; 95% CIs -1.14, -0.26; I^2^ = 48%; 4 trials, *n* = 175) (Fig. 2b). This finding is statistically significant with a moderate-large effect.

There is low quality evidence that supervised exercises are not better than non-supervised exercises in improving lumbar range of motion in the short-term (MD -0.27; 95% Cis -0.70, 0.16; I2 = 31%; 2 trials, *n* = 68) (Fig. 2c).

##### Comparison: supervised exercises vs advice

Five trials [22, 24, 32–34] reported results on the effect of supervised exercises vs clinical advice. The reviewers decided to use the term advice to collect interventions like guidebook, booklet or electronic file containing advice on exercises and movements to do or to avoid.

A high statistical heterogeneity emerged, partially reduced with subgroup analysis: in three trials [32–34] the treatment started early after surgery, while in two trials [22, 24] a delayed rehabilitation approach was delivered.

There is very low quality evidence that supervised exercises are better than advice in reducing pain (SMD -0.91; 95% CIs -1.61, -0.21; I2 = 87%; 5 trials, *n* = 341) (Fig. 2d) and disability (SMD -0.80; 95% CIs -1.59, -0.01; I2 = 84%, 4 trials, *n* = 261) in the short-term (Fig. 2e). After the subgroup analyses, only the delayed approach confirmed these findings for both outcomes, while no significant differences emerged in the early approach.

##### Comparison: supervised exercises vs no treatment

Two trials [15, 29] reported results on the effect of supervised exercises vs no treatment with a late rehabilitation approach. A medium to low statistical heterogeneity was found.

There is very low-quality evidence supporting that supervised exercises are better than treatment in reducing pain (MD -0.34; 95% CIs -7.32, 6.63; I2 = 5%; 2 trials, *n* = 166) (Fig. 2f) and disability (SMD -0.13; 95% CIs -0.5, 0.24; I2 = 11%; 2 trials, *n* = 166) (Fig. 2g) at 3 months after intervention.

There is very low-quality evidence that no treatment is better than supervised exercises in reducing pain (MD 9.28; 95% CIs 2.78, 15.77; I2 = 9%; 2 trials, *n* = 166) (Fig. 2h) and no better than supervised exercises for disability (SMD -0.06; 95% CIs -0.71, 0.59; I2 = 53%; 2 trials, *n* = 166) (Fig. 2i) at 6 months after surgery.

#### Qualitative analysis

A complete description of the qualitative analysis of the lumbar disc herniation studies is reported in Additional file 3.

#### Adverse events

No relevant adverse events or further surgery related to the post-surgical rehabilitation emerged.

### Assessment of risk of bias: overview

Only 8 included studies had low risk of bias [19, 20, 22, 23, 25, 34, 35, 37, 43], whereas 14 studies had moderate risk of bias [14, 18, 28, 31, 33, 38, 39, 41, 47, 53–55, 58, 59], and 21 studies had high risk of bias [15, 16, 24, 26, 27, 29, 30, 32, 36, 40, 42, 44–46, 48–52, 56, 57] (see Table 2).

The randomization procedure and allocation concealment were not adequately described in 13 RCTs with a high risk of bias and in 8 articles with a moderate risk of bias.

Deviations from the interventions occurred in 27 studies, mainly due to the inability to blind patients and caregivers to the interventions. 10 articles did not report all data, so results were probably biased by missing information.

In 13 RCTs with a high risk of bias and 5 RCTs with a moderate risk of bias, the authors used an inadequate method to detect outcomes between groups. For example Beneck et al. [50] used a patient-reported outcome measure, but only patients in the intervention group had a constant confrontation with the therapist, and this may have influenced the final questionnaire score. Finally, only 2 RCTs presented problems in the selection of the reported result; for example one RCT [49] did not report secondary outcome scores related to disability and pain.

## Discussion

The aim of this systematic review was to investigate the effectiveness of postoperative physiotherapy, in patients after surgery for lumbar stenosis, spondylolisthesis, or disc herniation. Due to the scarcity and heterogeneity of data collected, quantitative analysis was conducted only for the population with lumbar disc herniation. In fact, not enough articles were found on stenosis and spondylolisthesis populations, and most of them considered mixed samples without stratification by pathology. Finally, the outcomes and endpoints were often heterogeneous and not comparable.

Concerning lumbar stenosis, the article by Chen et al. [18] showed that a supervised active exercise program brings some improvements in pain, disability, and quality of life (mental health) in both the short and medium term, while no significant improvements were observed in functional testing. The results of this study are consistent with a Cochrane review [10], reporting the effectiveness of active exercise, in patients operated for lumbar stenosis.

Only one study on lumbar stenosis could be included in this review, while three were included in the Cochrane review [10], where a meta-analysis was performed. One reason for this difference is related to our inclusion criteria, which were more stringent. In particular, the study by Mannion et al. [1], included in the Cochrane review, was excluded from our work because it considered patients who had previously undergone lumbar surgery; the study by Aalto et al. [61] was excluded due to the lack of a true randomization. In addition, Cochrane authors [10] included in their meta-analysis two studies on heterogeneous populations, which did not meet specific diagnostic criteria for lumbar spinal stenosis. Indeed, both studies by Mannion et al. [1] and McGregor et al. [31] also included also patients operated for herniated discs.

The three articles on spondylolisthesis included in our review [19–21] were considered as one study. In the first publication [19], there was no significant difference on disability between the home exercises and the usual care group; the first ODI assessment was performed three months after surgery, when the exercise intervention started. This is the reason why it was not possible to measure the change in disability during the early three months after surgery, as in the study by Abbott et al. [60] where this outcome significantly improved. However, Abbott studied a mixed population of patients operated for spondylolisthesis, herniated disc, and stenosis. Information and education strategies provided only to the exercise group did not seem to have the same effect as in the study by Monticone et al. [62] on a mixed population (patients operated for spondylolisthesis and stenosis), in which cognitive and behavioral treatment in addition to exercise was more effective than exercise alone.

Kinesiophobia and activity levels assessed by Ilves et al. [20] did not significantly change between the two groups, despite the presence of educational sessions in the intervention group compared to the control one. Like disability, the greatest improvement of kinesiophobia seems to occur in the first months after surgery.

These results could suggest to investigate the best timing of rehabilitation program, as Oestergaard et al. [63, 64] found that early rehabilitation had no significant ineffective on pain, disability, and quality of life, and it was more expensive. There is currently no consensus in the literature on the most effective rehabilitation program after surgery for lumbar spondylolisthesis.

Concerning physiotherapy after surgery for lumbar herniation, our findings support the Cochrane review by Oosterhuis et al. [8], which suggested that therapeutic programs should be started between week 4 and 6 after surgery and that the methodological quality of future RCT studies should be improved. However, there are differences in the inclusion criteria between the present study and the Cochrane review. In fact, we excluded the studies by Alaranta et al. [65], Kjellby-Wendt et al. [66] and Donaldson et al. [67] because they included patients younger than 18 years old; the study of Scrimshaw et al. [68] due to a mixed population considering patients with herniated disc, stenosis, and spondylolisthesis; and finally, the study by Timm et al. [69] which was performed on a sample that was not representative of the general population, as all subjects worked in the same place.

We found a relevant heterogeneity in terms of type, duration, frequency, and intensity of rehabilitation interventions. However, it was noted that in 28 studies [14, 22–26, 28, 32–39, 42–44, 46, 48, 49, 51–55, 57, 59], rehabilitation started within four weeks after surgery, while in 12 studies [15, 16, 22, 26, 29, 31, 41, 45, 47, 50, 56, 58], physiotherapy began after the fourth postoperative week. Only one study [39] considered a treatment starting one year after surgery.

Compared to the systematic review by Atsidakou et al. [70] which support with moderate evidence the effectiveness of an early rehabilitation in patients operated for lumbar disc herniation, the results of our systematic review argues for greater caution. This different conclusion is probably related to the fact that Atsidakou et al. [70] included only seven RCTs, excluding all studies on patients performing supervised exercise as a control group or studies in which the intervention group carried out any other treatment, besides supervised exercise.

Regarding the type of intervention, most of the supervised exercises were described, although the programs were very heterogeneous. It should be noted that in some studies [14, 16, 22–24, 26–29, 31, 32, 34–39, 42, 44, 46, 51, 55, 57–59] the authors described the details and dosage of each exercise, while others did not reported them [15, 21, 22, 25, 31, 40, 41, 43, 45, 47–49, 52–54]. Extracting and categorizing treatments for both intervention and control groups was very difficult because of the reporting of the articles and the high heterogeneity of interventions’ types and definitions (e.g. “usual care”, “no treatment”, “advice”, etc.). We strongly expect that future RCTs follow the TIDieR Checklist and the World Physiotherapy glossary for defining and reporting interventions [71].

After performing the meta-analyses, a clear direction of effect can be confirmed toward a face-to-face physiotherapy intervention versus non-supervised exercises or post-surgical pain and disability counseling for the delayed approach. However, an early approach should not be discouraged a priori, but more primary studies investigating the immediate post-surgery rehabilitation are needed.

In a different way from the Cochrane Review by Oosterhuis et al. [8], our findings suggest a significant improvement in disability for patients undergoing supervised exercise programs compared with those involved in non-supervised exercise programs; this discordance is mostly due to the inclusion of two RCTs [16, 28] published after the last search by the Cochrane group.

Education as an intervention has been studied in many articles. According to Ostelo et al. [47], different educational interventions (cognitive-behavioral therapy and standard education) produce similar effects, while the combination of supervised exercises with education can produce superior results, compared to instruction alone [30, 50, 51].

A limitation in this review may be the exclusion of grey literature consistently with our protocol.

## Conclusions

In this review only studies concerning rehabilitation after surgery for lumbar disc herniation could be pooled in a quantitative analysis. These meta-analyses showed with low quality evidence that supervised exercises can reduce short-term pain and disability compared with non-supervised exercises, but do not significantly improve lumbar range of motion. In addition, supervised exercises were found to be better than advice in reducing short-term pain and disability, with a low quality evidence.

Suggestions concerning rehabilitation after lumbar spinal stenosis and spondylolisthesis surgery arise only from qualitative analyses. In lumbar spinal stenosis surgery, education and exercises in the peri-surgical period seems to induce similar results when compared to usual care. In lumbar spondylolisthesis surgery, home exercises significantly reduce kinesiophobia in the short-term, but do not improve pain or disability in the long-term follow-ups, compared with usual care.

This research suggests that multimodal rehabilitation protocols after lumbar surgery may be used to improve outcomes such as pain, disability, and physical function, but reveals a scarcity of RCTs regarding rehabilitation after surgery for lumbar spinal stenosis and lumbar spondylolisthesis and relevant heterogeneity of proposed interventions.

Further research should be conducted on the effectiveness of combined pre-operative and post-operative rehabilitation programs, the best time to start physiotherapy after interventions, and the long-term effects. There is a need to improve the overall quality of the studies on this topic.

### Supplementary Information

**Additional file 1.** 

**Additional file 2.** 

**Additional file 3.**

## Data Availability

The datasets analysed during the current review and the metanalisis are available from the correspondent author on reasonable request.

## Abbreviations

CIs: Confidence Intervals
EQ-5D: EuroQol-5 Dimension
GRADE: Granding of recommendation, Assessment, development and Evaluation
LBP: Low Back Pain
MD: Mean Difference
NASS: North American Spine Society
NRS: Numeric rating Scale
ODI: Oswestry Disability Index
PICO: Patient, Intervention/Treatment, Control, Outcome
RCT: Randomized Clinical Trial
RoB: Risk of Bias
SF-36: Short-Form 36
SMD: Standardized Mean Difference
SoF: Summary of Findings
TIDieR: Template for Intervention Description and Replication
VAS: Visual Analogue Scale
WHO: World Health Organization
