# Supplementary material for: Rehabilitation after lumbar spine surgery in adults: a systematic review with meta-analysis
Source: Arch Physiother. 2023 Oct 16;13:21. doi: 10.1186/s40945-023-00175-4 (PMC10578022; doi:10.1186/s40945-023-00175-4)
Supplement: Supplementary file 1 — Additional file 1. [file 40945_2023_175_MOESM1_ESM.pdf]

Appendix 1 - MEDLINE search strategy

Search number	Query	Search number	Query
1	spinal stenos* [Title/Abstract]	36	laser therap* [MeSH Terms]
2	spinal stenos* [MeSH Terms]	37	magnetotherapy [Title/Abstract]
3	,spinal stenos*	38	TENS [Title/Abstract]
4	spondylolisthes* [Title/Abstract]	39	dry needling [Title/Abstract]
5	lumbar stenos* [Title/Abstract]	40	acupuncture [MeSH Terms]
6	foraminal stenos* [Title/Abstract]	41	neurodynamic* [Title/Abstract]
7	lumbar spondylolisthes* [Title/Abstract]	42	neural mobilization [Title/Abstract]
8	vertebral spondylolisthes* [Title/Abstract]	43	nerve gliding [Title/Abstract]
9	degeneration, intervetebral disc [MeSH Terms]	44	hyperthermia, induced [Title/Abstract]
10	intervetebral dis* [MeSH Terms]	45	massage* [Title/Abstract]
11	disc displacement, intervetebral [MeSH Terms]	46	mobilitation* [Title/Abstract]
12	disc displacement [MeSH Terms]	47	othotic* [Title/Abstract]
13	lumbar disc degeneration [Title/Abstract]	48	treadmill* [Title/Abstract]
14	lumbar disc displacement [Title/Abstract]	49	walk* [Title/Abstract]
15	spinal disease [MeSH Terms]	50	cycl* [Title/Abstract]
16	**OR #1 - #15**	51	bicycl* [Title/Abstract]
17	exercise therap* [MeSH Terms]	52	training* [Title/Abstract]
18	rehabilitation [MeSH Terms]	53	physical activit* [MeSH Terms]
19	rehab* [MeSH Terms]	54	postoperative rehabilitation* [Title/Abstract]
20	physical and rehabilitation therapy [MeSH Terms]	55	Core stabilit* [Title/Abstract]
21	cognitive behavioral therapy [Title/Abstract]	56	McKenzie [Title/Abstract]
22	cognitive-behavioral therapy treatment [Title/Abstract]	57	neuromuscular training* [Title/Abstract]
23	physical therapy [Title/Abstract]	58	**OR #17 - #57**
24	manual therapy [Title/Abstract]	59	discectomy [MeSH Terms]
25	physiotherapies techniques [MeSH Terms]	60	laminectomy [MeSH Terms]
26	health education [MeSH Terms]	61	microdiscectomy [Title/Abstract]
27	aftercare [MeSH Terms]	62	root decompression [Title/Abstract]
28	physical education [MeSH Terms]	63	nerve decompression [Title/Abstract]
29	traction [Title/Abstract]	64	lumbar decompression [Title/Abstract]
30	self management [MeSH Terms]	65	spinal fusion* [MeSH Terms]
31	manipulation [Title/Abstract]	66	surgical stabilization* [Title/Abstract]
32	adjustment, chiropractic spinal [MeSH Terms]	67	vertebral stabilization [Title/Abstract]
33	exercise, muscle stretching [MeSH Terms]	68	surgery [MeSH Terms]
34	stretching [Title/Abstract]	69	**OR #59 - #68**
35	taping [Title/Abstract]	70	**#16 AND #58 AND #69**
