# Supplementary material for: Rehabilitation after lumbar spine surgery in adults: a systematic review with meta-analysis
Source: Arch Physiother. 2023 Oct 16;13:21. doi: 10.1186/s40945-023-00175-4 (PMC10578022; doi:10.1186/s40945-023-00175-4)
Supplement: Supplementary file 2 — Additional file 2. [file 40945_2023_175_MOESM2_ESM.docx]

Additional file 2 – Characteristics of all studies included

| **LUMBAR SPINAL STENOSIS** | | | | | |  |
| --- | --- | --- | --- | --- | --- | --- |
|  | **Population** | **Stage of intervention** | **Description** | **Endpoint (weeks after surgery)** | **Outcomes** | **Results with effective intervention** |
| Chen et al. (15) | 60 patients  age 18-65    IG: 29  Mean age: 51,8 ± 11,6  Gender (M%): 55,2%  CG: 31  Mean age: 52,1 ±9,9  Gender (M%): 45,2% | early stage (immediately after surgery) | **IG: Supervised exercise + education**  Patient pre and post-operative education, mobilization strategies, core stability exercises.  30 min / day, for the entire duration of hospitalization.  **CG: usual care**  Instructions concerning post-operative care by the involved neurosurgical team, i.e., the usual care protocol. | 4weeks  12weeks  24weeks | RMDQ  SF12  VAS (leg and back pain)  Global rating scale  Functional capacity (Forward reaching, 5 repeated sit to stands, Timed 15-m walk, 1 minute going up and down stairs) | 4 weeks. IG demonstrated significant improvement at VAS and RMDQ respect CG  12-24weeks. No significant differences between the two groups were found regarding all outcomes |
| **SPONDYLOLISTHESIS** | | | | | |  |
|  | **Population** | **Stage of intervention** | **Intervention** | **Endpoint (weeks after surgery)** | **Outcomes** | **Results with effective intervention** |
| Ilves et al. (16) | 98 patients  IG: 48  mean age: 59 ± 12  Gender (M%): 29%  CG: 50  mean age: 58 ± 12  Gender (M%): 24% | Late phase: 3 months after surgery. | **IG: Supervised + unsupervised exercise**  Specific exercise program to be performed at home for a year, with recall sessions scheduled every two months, during which cognitive-behavioral advice was also given.  **CG: Usual care**  Oral and written instructions given to the patient in a single session, without any prompts or progression of difficulty in the exercises. | 64 weeks  116 weeks | ODI    RAND-36  . | 64-116 weeks. No significant differences between the two groups were found regarding all outcomes |
| Ilves et al. (17) | 98 patients  IG: 48  mean age: 59 ± 12  Gender (M%): 29%  CG: 50  mean age: 58 ± 12  Gender (M%): 24% | Late phase: 3 months after surgery. | **IG: Supervised + unsupervised exercise**  **CG: Usual care** | 116 weeks | Tampa Scale for Kinesiophobia  International Physical Activity Questionnaire  VAS (leg and back pain) | 116 weeks. No significant differences between the two groups were found regarding all outcomes |
| Ilves et al (18) | 98 patients  IG: 48  mean age: 59 ± 12  Gender (M%): 29%  CG: 50  mean age: 58 ± 12  Gender (M%): 24% | Late phase: 3 months after surgery. | **IG: Supervised + unsupervised exercise**  **CG: Usual care** | 116 weeks | Trunk Isometric strength (strain-gauge dynamometer + computer program)  Trunk endurance (Biering-Sørensen’s static hold test)  VAS (leg and back pain)  Lumbar ROM  Timed Up and Go test | 116 weeks. No significant differences between the two groups were found regarding all outcomes |
| **DISC HERNIATION** | | | | | |  |
|  | **Population** | **Stage of intervention** | **Intervention** | **Endpoint (weeks after surgery)** | **Outcome** | **Results with effective intervention** |
| Lu et al. (19) | 60 patients  IG: 30  mean age: 48.5 ±16.5  Gender (M%):  CG: 30  mean age: 50.2 ± 14.0  Gender (M%): | early stage | **IG: supervised exercise** (McKenzie technique)  The experimental group performed remote phase rehabilitation guidance based on McKenzie technique: 2-6 weeks after the operation as the first phase, 7-12 weeks as the second phase, and 13-24 weeks as a third phase.  **CG: unsupervised exercise**  The control group received a booklet with exercises | 6 weeks  12 weeks  24 weeks | VAS  ODI  SF-36  JOA | 6 weeks. IG demonstrated significant improvement at VAS, JOA, SF-36 and ODI respect CG.  12 weeks. IG demonstrated significant improvement at VAS, SF-36 and ODI respect CG.  24 weeks. IG demonstrated significant improvement at SF-36 respect CG. |
| Johansson et al. (20) | 59 patients  IG: 29  Mean age: 39.2 ±9.4  Gender (M%): 59%  CG: 30  Mean age: 42.7±10.1  Gender (M%): 60% | early stage (first day after surgery for both group whereas the different training – IG and CG - started 12 weeks after surgery) | Both groups: first day after surgery all patients started stabilization of the back and hip mobility, activation back, abdominal and buttock muscles, transfers. A written exercise program to follow at least once a day. The equal program finished after 3 weeks  **IG: supervise exercise**  They worked on their exercises under supervision by the physiotherapist alongside their daily home program, including graded activity with positive reinforcement.  Eight weeks, 1/wk.  **CG: unsupervised exercise**  Same exercise program but to be performed at home as mentioned gradually increase the number of repetitions | 12 weeks  48 weeks | VAS (leg and back pain)  ODI  TSK (12-48)  CSQ (self-statement) 0-36  CSQ (Catastrophizing) 0-36  EuroQoL 5D  EuroQoL VAS (0-100) | 12 weeks. No significant differences between the two groups were found regarding all outcomes  48 weeks. CG demonstrated significant improvement at VAS (back pain) and at quality of life respect CG;  IG demonstrated significantly improvement at activity levels and more satisfied respect CG |
| Hebert et al. (21) | 61 patients  age 18-60  IG: 29  Mean age: 40,6 ± 10, 2  Gender (M%): 44,8%  CG: 32  mean age: 40,2 ± 8,8  Gender (M%): 53% | early stage (2 weeks after surgery) | **IG: supervised exercise program +** specific trunk exercise for 8 weeks  **CG: supervised exercise program** without specific trunk exercise for 8 weeks | 10 weeks  24 weeks (by email or phone call) | ODI  NRS 0-10 (leg and back pain)  Sciatica Frequency and Sciatica Bothersomeness (0–25 score)  Muscle thickness change on side of surgery (%)  Muscle thickness change contralateral to surgery (%)  Global rating of change | 10-24 weeks. No significant differences between the two groups were found regarding all outcomes |
| Zhang et al. (22) | 92 patients  age 20-68  Mean age:57.4±6.1  IG: 46  Mean age and Gender (M%):not specified*  CG: 46  Mean age and Gender (M%):not specified*  * In the article it is reported: “The general information of the two groups had no  Difference through comparison (p>0.05)”. | early stage (1 day after surgery) | **IG: supervised exercise**  Early functional exercises of passive and autonomic activities, including extension and flexion exercises of the lower limbs, toes and neck, functional exercises of the back muscles. After a full recovery, patients could continue to perform functional exercises for more than 60 min every day.  **CG: unsupervised exercise**  The control group performed routine functional exercises after their operations. | 12 weeks  24 weeks | SF-36  Lumbar function scale score (residual lumbo-crural pain, SLR, muscle strength, sensory and nerve reflex) | 12 weeks. IG demonstrated significant improvement at residual lumbo-crural pain, SRL, muscle strength; total score of lumbar function respect CG  24 weeks. IG demonstrated significant improvement at PF SF-36, Activity SF-36, SF SF-36, MH SF-36, QoL SF-36 respect CG. No significant differences in EF SF-36 |
| Danielsen et al. (23) | 63 patients  age 20 - 60  IG: 39  Mean age: 37,9  Gender (M%): 61,50%  CG: 24  mean age: 42,4  Gender (M%): 70,80% | late stage (4 weeks after surgery) | **IG: supervised exercise** (intensive program)  supervised active rehabilitation program including a regimen of vigorous lumbar stabilizing exercises 3 times/week (40 minutes a session)  **CG: unsupervised exercise** (mild program)  Mild program of 2 to 3 back exercises at home, after relaxing and resting their backs for 2 months after the surgery. | 24 weeks  48 weeks | VAS  RMDQ  ADL Participation (as usual or almost without difficulties/with some or great difficulties)  Return to work (follow up 6- 12 months) | 24 weeks. IG demonstrated a significant improvement at RMDQ, VAS and in the self-evaluated health respect CG  48 weeks. IG registered a significant improvement at RMDQ , VAS and in the self-evaluated health respect CG |
| Demir et al. (24) | 44 patients  age 20-65  IG: 22  Mean age: 43.0 ± 8.2  Gender (M%): 50%  CG: 22  mean age: 39.2 ±9.6  Gender (M%):59.1 % | late stage (4 weeks after surgery) | **IG supervised exercise.**  Dynamic lumbar stabilization exercises (three days a week) + Home-based exercises program  **CG: unsupervised exercise**  The home exercise program prescribed to both groups consisted of stretching, pelvic tilt, flexion and extension strengthening of the abdomen and the trunk (45 minutes, once every day, with ten repetitions each exercise) | 4 weeks  8weeks  24 weeks | ODI  VAS (back and leg pain)  FABQ  MLS (modified Schober test)  LS (lumber schober test)  FFD (Finger -Foot Distance)  Right and Left LF (lateral flexion) | 4 weeks. No significant differences between the two groups were found regarding all outcomes  8 weeks. IG demonstrated significant improvement at all the outcomes respect CG.  24 weeks. The improvements at 8weeks remained until 24weeks measurement. |
| Yilmaz et al. (25) | 42 patients  age 22 - 60  IG: 14  Mean age: 46 ±9,77  Gender (M%): 57%  IG2: 14  mean age: 41 ±8.88  Gender (M%): 43%    CG: 14  mean age: 42,79 ± 11,39  Gender (M%): 57% | late stage (4 weeks after surgery) | **IG: supervised exercise**  Dynamic lumbar stabilization exercise for eight weeks under supervision; 3 days/week  **IG2: unsupervised exercise**  Flexion‐extension program (Williams‐McKenzie) home program for eight weeks  **CG: advice** | 12 weeks | VAS (back pain)  Modified Oswestry Disability Index  Beck Depression Scale  Finger floor distance  Lumbar Schöber  Modified lumbar Schöber  Progressive Isoinertial Lifting Evaluation (PILE) | 12 weeks. IG demonstrated significant improvement in all outcomes.  IG2 demonstrated improvement at VAS, MOI, LS and PILE. |
| Choi et al. (26) | 75 patients, mean age 46.09 years  IG: 35  Mean age: 51.05 ± 9.58  Gender (M%): 57,14%  CG: 40  mean age:42.02 ± 17.06  Gender (M%): 45% | Late stage (IG: 6 weeks after surgery) | Both groups: on discharge all patients were given advice on maintaining proper posture and avoiding strenuous activities and a home exercise program (for 6 weeks)  **IG: supervised exercise**  The intervention group began an intensive program with a defined set of strengthening exercises for the extensor muscles (MedX system). Aerobic and stretching exercises were also included.  **CG: unsupervised exercise**  The control group continued with the home-based basic lumbar conditioning exercises. | 18 weeks  24 weeks  48 weeks | ODI  VAS  Trunk extensor strength (with the MEDX)  Return to work | 18 weeks. IG demonstrated significant differences at VAS and Trunk extensor strength respect CG.  24 weeks. IG demonstrated significant differences at return to work outcome ( faster return to work) respect CG  48weeks. No significant differences between the two groups were found regarding all outcomes |
| Filiz et al. (27) | 60 patients  G: 20  Mean age: 38,2 ± 1,54  Gender (M%): 50%  IG: 20  mean age: 41,25 ± 1,18  Gender (M%): 60%  CG: 20 | late stage (4 weeks after surgery) | **IG: supervised exercise** (intensive program)  Intensive exercise program and back school education under supervision  8 weeks; 3 days a week with sessions of 1.5 hours each.  **IG2: unsupervised exercise**  This group learned the McKenzie and Williams exercises in the clinic and later did these exercises at home three days a week. The home exercise program  was followed up by telephoning the patients once a week.  **CG: advice** | 12weeks | Lumbar Schöber (LS)  VAS  Weight lifting capacity  Progressive Isoinertial Lifting Evaluation (PILE)  Back endurance, abdominal endurance  Modified ODI  Beck Depression Inventory  Low Back Pain Rating Scale  Return to work | 12 weeks. IG and IG2 demonstrated significant differences at LS, Modified ODI, Back and abdominal endurance respect CG  IG demonstrated significant difference at VAS and return to work outcome more than IG2 and CG  IG demonstrated significant improvement for BDI respect CG |
| Dolan et al. (28) | 20 patients  age 18-60  Gender (M%): 86%  IG: 9  Mean age: 39.2 ±9.4  Gender (M%): not specified  CG: 11  Mean age: 42.7 ±10.1  Gender (M%): not specified | late stage (6 weeks after surgery) | **IG: supervised exercise**  General aerobic exercises, stretching exercises, extension exercises, strength and endurance exercises (back and abdominal). 60 min, 2 times/week (for 4 weeks)  **CG: no treatment** | 6 weeks  10 weeks  26 weeks  54 weeks | VAS  Low-Back Outcome Score  Multidimensional health locus of Control (HLC)  Modified Somatic Perception Questionnaire (MSPQ)  Zung Depression Scale (ZDS)  Posture and Mobility of spine (with 3-Space Isotrack)  Muscle fatigue (with electromyography) | 54 weeks. IG demonstrated significant improvement for VAS, Low-back outcome score respect CG |
| Paulsen et al. (29) | 146 patients  age 18-65  IG: 73  Mean age: 42.9 ± 8.9  Gender (M%): 63%  CG: 73  mean age: 42.8 ± 11.8  Gender (M%): 63% | late stage (4-6 weeks after surgery) | Both groups: prior to surgery, both groups had performed standard exercises in the first 4 weeks post-surgery and they got booklets and recommendations.  **IG: supervised exercise**  4-6 weeks after surgery the IG received municipal rehabilitation referral (exercises for spinal stability, the exact type of exercise may vary).  1-2 times/week, 8-10 weeks  **CG: no treatment**  4-6 weeks after surgery the control group don’t have any other scheduled physical therapy visits or referrals | 4 weeks  12 weeks  24 weeks  48 weeks  96 weeks | ODI  EuroQoL-5D  VAS (leg and back pain) | No significant differences between the two groups were found regarding all outcomes at all endpoints |
| Kulig et al. (30) | 98 patients  age 18 - 60  IG: 51  mean age: 39.2 ±10.2  Gender (M%): 58%  CG: 47  mean age: 41.4 ± 9.9  Gender (M%): 50% | late stage (4-6 weeks after surgery) | **IG: education + supervised exercise**  One‐session back care education + 12- week Spine Exercise Program for Back extensor strength and endurance training  **CG: education**  One‐session back care education  **UPT: usual physical therapy**  after their allocation, some  of the participants self-selected a  course of physical therapy at a clinic  of their choosing | 12 weeks | ODI  Repeated sit to stand test  50 foot Walk test,  5 minutes’ walk test | *(post-hoc comparison)*  12 weeks. IG demonstrated significant improvement for ODI, 5 minute walking test, 50 foot walk test respect CG |
| McGregor, et al. (31) | 338 patients  age >18 years  IG1: 86  mean age: 54 ±15  Gender (M%): 43%  IG2: 91  mean age: 53±15  Gender (M%): 52%  IG3: 70  mean age: 53 ±15  Gender (M%): 51%  CG: 91  mean age: 55 ±16  Gender (M%): 43% | late stage (6-8 weeks after surgery) | **IG1: supervised exercise**  12 one‐hour classes, aerobic fitness; stretching; stability exercises; strengthening and endurance training for the back, abdominal, and leg muscles; ergonomic training; advice on lifting and setting targets; and self-motivation.  12 sessions 2 times/week (1 hour session)  **IG2: supervised exercise + education**  Same program and educational booklet ‘Your Back Operation’  **IG3: education**  The patients received only the educational booklet "Your Back Operation"  **CG: usual care**  Patients receiving usual care were managed according to the relevant surgeon’s usual practice | 12 weeks | ODI  VAS (leg and back pain)  HADS  FABQ  VAS health summary | 12 weeks. No significant differences between the four groups regarding all outcomes. |
| Oosterhuis et al. (32) | 169  age 18-70  IG: 92  Mean age: 47 ±12  Gender (M%): 41%  CG: 77  mean age: 47 ±12  Gender (M%): 43% | early stage (1 week after surgery) | Both group: during hospitalization all patients received instructions for transfers and activities of daily living. At discharge, all patients received leaflet with advice, and suggestions on exercises.    **IG: supervised exercise + advice + unsupervised exercise**  Supervised exercise starting the first week after discharge. Over 6 to 8 weeks, participants received one or two individual, face-to-face, exercise therapy sessions of 30 minutes per week.  **CG: advice + unsupervised exercise** | 3 weeks  6 weeks  9 weeks  12 weeks  26 weeks | ODI  NRS (leg and back pain)  SF12 | No significant differences were found between the two groups regarding all outcomes at any endpoints |
| Janssens et al. (33) | 25 patients age 18-60  IG: 12  Mean age: 46 ± 11  Gender (M%):  CG:13  mean age: 46 ± 8  Gender (M%): | early stage (2 weeks after surgery) | **IG1: supervised exercise**  Patient education, ergonomics exercises, motor control exercises. If necessary, segmental spinal mobilization and neurodynamics (active and passive slider techniques of the sciatic nerve).  8-15 physiotherapy sessions for 12 weeks.  **CG: advice**  Basic ergonomic advice, the advice to stay active and a restriction of physiotherapy for at least 12 weeks. | 8 weeks  24 weeks  52 weeks | NRS  ODI  Tampa Scale of Kinesiophobia (TSK)  Global perceived effect  Work absence  Relative Proprioceptive Weighting (RPW)  Sit-to-stand-to sit movements (STSTS) | 8 weeks. IG demonstrated significant improvement for RPW, STSTS respect CG  24 weeks. IG demonstrated significant improvement for RPW respect CG  52 weeks. IG demonstrated significant improvement for TSK and work absence respect CG |
| Erdogmus, et al. (34) | 120 patients  IG: 40  Mean age: 39,8 ± 10,5  Gender (M%): 52,5%  CG: 40  mean age: 42,3 ± 9,8  Gender (M%): 52,5%  CG2: 40  mean age: 41,8 ± 10,4  Gender (M%): 62,5% | early stage (1 week after surgery) | **IG: supervised exercise**  Strength exercises, stretching, ergonomics, improvement in general mobility of the spine, improving muscle coordination and automatic muscle response time.  12 weeks, 20 sessions of 30 minutes  **CG1: sham treatment**  20 sessions “sham” neck massage of 30 minutes duration.  **CG2: advice**  “wait and see” for the first three months after operation, and no particular treatment was planned (they could, however, have received any kind of treatment their family doctor deemed appropriate or necessary) | 6 weeks  12 weeks  78weeks | Low Back Pain Rating Scale (LBP-RS)  Likert scale  Return to work  State Trait Anxiety Inventory (STAI)  Use of analgesics | 6 weeks. No significant differences were found between the three groups  12 weeks. IG demonstrated significant improvement for LBP-RS respect CG2. No significant differences were found between IG e CG1 and between CG1 and CG2  78 weeks. IG demonstrated significant reduction at use of analgesics respect CG2. No significant differences were found between IG e CG1 and between CG1 and CG2 |
| Ebenbichler et al. (35) | 74 patients  IG: 29  Mean age:  Gender (M%):  CG: 22  mean age:  Gender (M%):  CG2: 23  mean age:  Gender (M%): | early stage (1 week after surgery) | **IG: supervised exercise**  Strength exercises, stretching, ergonomics, improvement in general mobility of the spine, improving muscle coordination and automatic muscle response time.  12 weeks, 20 sessions of 30 minutes  **CG1: sham treatment**  20 sessions “sham” neck massage of 30 minutes duration.  **CG2: advice**  “wait and see” for the first three months after operation, and no particular treatment was planned | 624 weeks (=12 years) | Low Back Pain Rating Scale (LBP-RS)  Likert scale  Beck depression Inventory score (BDIS) | 624 weeks. IG demonstrated significant improvement for LBP-R, Back Complaints and BDIS respect CG2. No significant differences were found between IG e CG1 and between CG1 and CG2 |
| Ju et al. (36) | 14 patients  IG: 7  Mean age: 45.2 ±3.96  Gender (M%):  CG: 7  mean age: 46.2 ± 5.3  Gender (M%): | early stage | **IG: supervised exercise.**  The program consisted of the Medx lumbar extension program and progressive resistance exercise.  three times/week, 70 minutes per session  **CG: no treatment** | 12 weeks | Isometric strength (Medx) at 0°-24°-48°-72° lumbar angles flexion  VAS (back pain, night pain, exercise pain, handicap) | 12 weeks. IG demonstrated significant improvement for VAS-exercise pain, VAS-handicap, extensor muscle strength at all angles |
| Kim et al. (37) | 33 patients  age 25-65  IG: 16  Mean age: 46,4 ±12,3  Gender (M%): 38%  CG: 17  mean age: 46,6 ± 11,9  Gender (M%): 29% | early stage (2-3 weeks after surgery) | **IG: manual therapy**  Osteopathic manipulative treatment. The protocol included manipulative and non-manipulative techniques without include high-velocity, low-amplitude thrust (HVLAT) manipulation of the lumbar segments where the surgery was performed. Eight individualized sessions, 30 min twice a week.  **CG: supervised exercise**  Active exercises program to improve spinal mobility and stabilize the lumbar segments increasing intensity and difficulty. Eight individualized sessions, 30 min twice a week. | 7-8 weeks | Roland and Morris Disability Questionnaire (RMDQ)  VAS (leg and back pain)  ROM with double inclinometer  Medication use | 7-8 weeks. IG demonstrated significant improvement for RMDQ respect CG |
| Kim et al. (38) | 21 patients  age 25-69  IG: 14  Mean age: 45,7 ±12,4  Gender (M%): 36%  CG: 7  mean age: 54,9 ±6,7  Gender (M%):71% | early stage (2-3 weeks after surgery). | **IG: manual therapy**  Osteopathic manipulative treatment. The protocol included manipulative and non-manipulative techniques without include high-velocity, low-amplitude thrust (HVLAT) manipulation of the lumbar segments where the surgery was performed. Eight individualized sessions, 30 min twice a week.  **CG: unsupervised exercise**  Home exercise program with booklet and verbal instruction. The active home exercise was recommended to perform twice a week for four weeks, each for half an hour | 7-8 weeks | VAS (0 -100) (leg and back pain)  RMDQ (0-24)  SF-36  Medication use | 7-8 weeks. IG demonstrated significant improvement for RMDQ , VAS (leg pain) and medication use respect CG |
| Häkkinen et al. (39) | 126 patients  IG: 65  Mean age: 39 ±7  Gender (M%):  CG: 61  mean age: 39 ± 8  Gender (M%): | late stage (2 months after surgery) | **IG: unsupervised exercise** (strength training)  Home strength training program for 12 months. Each exercise was practiced under supervision for 1 session.  2 series per exercise twice a week.  Instructions for stretching (3 times/week) and stabilization exercises (twice/week).  **CG: unsupervised exercise**  Only instructions for stretching (3 times/week) and stabilization exercises (twice/week). | 48 weeks | VAS (leg and back pain)  ODI  Million index  Isometric strength (Trunk flexion, Trunk extension)  Endurance (Trunk flexion, Trunk extension, squat)  Schöber test | 48 weeks. No significant differences between the two groups regarding all outcomes |
| Manniche et al. (40) | 96 patients  age 18-70  IG: 35  mean age:  CG: 34  mean age: | late stage (5 weeks after surgery) | **IG: supervised exercise** (Intensive exercises program)  Leg lifting, trunk lifting, abdominal exercise, leg abduction, leg adduction, bicycle training, stretching exercises. The patients were encouraged to continue the exercises independently from pain.  **CG: supervised exercise** (mild general mobilization exercises).  Hot water training pool and mobility exercises to the gymnasium  The patients were told to stop the exercises if pain or inconveniences occurred. | 6 weeks  26 weeks  52 weeks | Low Back Pain Rating Scale (LBP-RS) | 6 weeks. No significant differences between the two groups regarding all outcomes  26 weeks. IG demonstrated statistical improvement for the disability index of LBP-RS respect CG  52 weeks. No significant differences between the two groups regarding all outcomes |
| Zoia et al. (41) | 54 patients  IG: 29  mean age: 44,7  Gender (M%): 55,20%  CG: 25  mean age: 45,6  Gender (M%); 52,00% | Late stage (4 weeks after surgery) | **IG: corset**  Adoption of a lumbar corset in the upright position during the first 4 weeks after surgery. All of the prescribed corsets were semirigid (i.e., with posterior flexible stays and abdominal straps).  **CG: no corset**  Patients in group B also progressively returned to daily activities but without corset adoption | 4 weeks | VAS  ODI  RMDQ (1 month, follow up 6 months) | 4 weeks. No significant differences between the two groups regarding all outcomes |
| Kara et al. (42) | 54 patients  G: 29  Mean age:  Gender (M%):  CG: 25  mean age:  Gender (M%): | early stage (few hours after surgery) | **IG: Patient-controlled analgesia (PCA) + TENS** TENS was administered twice for 30 to 40 minutes each time with a 3 to 4 hour rest interval between the TENS applications.  **CG: Patient-controlled analgesia (PCA)** | 2 days | PCA demands  VAS (morning/evening, rest/activity),  Timed up & go  Beck depression inventory | 2 days- IG demonstrated significant reduction at PCA demands respect CG |
| Bono et al. (43) | 108 patients  IG: 53  Mean age: 42 ±12,5  Gender (M%): 45,90%  CG:55  mean age: 44,6 ± 9,4  Gender (M%): 55,60% | early stage (immediately after surgery) | **IG: Post-operative restrictions to activities for 2 months**  **CG: Post-operative restrictions to activities for 6 months** | 8 weeks  24 weeks | VAS (leg and back pain)  ODI | 8 weeks and 24 weeks. No significant differences in ODI, VAS back, or VAS leg scores were detected at any of the time points between the IG and CG |
| Aldemir et al. (44) | 67 patients  G: 14  Mean age:  Gender (M%):  CG: 7  mean age:  Gender (M%): | early stage (3 weeks after surgery) | **IG: Pedometer-supported walking and telemonitoring.**  This program starts with a minimum of 10 min (approximately 1200 steps) of walking 4 days a week and continues with a minimum of 30 min of moderate intensity walking for a minimum of 5 days a week.  **CG: education**  face-to-face interview 20-30 min | 3 weeks  4 weeks  8 weeks  12 weeks | Short Form McGill pain Questionnaire (SF-MPQ),  Modified Oswestry Disability Index (mODI),  SF-36 | 3 weeks. No significant differences in SF-MPQ, mODI and SF-36  4 weeks. IG demonstrated significant improvement at Momentary pain severity of SF-MPQ respect CG  8 weeks. IG demonstrated significant improvement at Verbal pain severity and Momentary pain severity of SF-MPQ, mODI respect CG  12 weeks. IG demonstrated significant improvement at mODI respect CG |
| Rothhaupt et al. (45) | 32 patients  IG: 16  mean age: 52.8  Gender (M%): 81,25%  CG: 16  mean age: 57.6  Gender (M%): 62,5% | Early stage (the mean duration between surgery and hospitalization where treatment was performed was 19.2 days in the IG and 19.8 days in the CG) | **IG: hippotherapy**  Orthopedic Hourseback Riding Therapy (OHRT) 20 min, 3 times/week  **CG: thermal cure** | 12 weeks | McNab score (postoperative condition)  MMPI (negative Psychic predictors)  Postoperative work disablement  Analgesics Consumers | *(results illustrated by graphic)*  12 weeks. IG demonstrated significant improvement at McNAB score, MMPI and postoperative work disablement. |
| Reyes et al. (46) | 24 patients  age 18-60  IG: 12  Mean age: 39.0 ± 13.9  Gender (M%): 58,3%  CG: 12  mean age: 43.6 ± 2.6  Gender (M%): 75% | early stage (3-4 weeks after surgery) | **IG: supervised exercise + neural mobilization.**  Standard rehabilitation to relieve pain, improve endurance and strength of trunk muscles, lower limbs, and abdominal muscles. Exercises ranged from 3 to 4 series of 5 to 10 repetitions.  Neural mobilization: knee extension and ankle dorsiflexion and cervical extension to decrease the tension of neural tissue. These movements were rhythmically and continuously performed for three minutes, divided into three sets of one minute.  2-3 times/week (10 sessions).  **CG: supervised exercise + TENS**  Same standard rehabilitation program of the IG.  Patients also received TENS combined with superficial thermotherapy (i.e., hot packs) on the lumbar area. | 6 weeks | VAS (leg and back pain)  ODI  SF-36 | 6 weeks. No significant differences were found between the two groups regarding all the outcomes. |
| Ostelo et al. (47) | 105 patients  age 18-65  IG: 52  Mean age: 42,8 ± 8,8  Gender (M%): 50%  CG: 53  mean age: 43,7 ± 8,8  Gender (M%): 64,2% | early stage | **IG: Behavioural Graded Activity (BGA)**  Individually tailored exercise program of increasing intensity, behavioral graded activity (operant therapy) using graded activity and positive reinforcement, time‐contingency management.  30 min/session over a three‐month period (max 18 sessions)  **CG: usual care**  The whole spectrum of techniques used by physiotherapists were included, but cognitive behavioral treatment, acupuncture, osteopathic techniques and all kinds of other “alternative” techniques were excluded.  30 min/session over a three‐month period (max 18 sessions) | 12 weeks | Global Perceived Effect with the 7 point scale  RMDQ  Pain Catastrophizing Scale (PCS)  Tampa Scale for Kinesiophobia (TSK)  VAS (0-100) (leg and back pain)  Main Complaints (two ADL chosen by the patient)  SF-36 lumbar  ROM with the Cybex edi-320 | 12 weeks. No significant differences were found between the two groups regarding all the outcomes |
| Kacar et al. (48) | 20 patients  IG: 10  Mean age: 39.8 ± 8.2  Gender (M%): 60%  CG: 10  mean age: 44.0 ± 8.6  Gender (M%): 30% | early stage (2 weeks after surgery) | **IG: Exercises using pressure biofeedback**  **3 times/weeks for 5 weeks**  (15 sessions)  **CG: Normal exercises program without biofeedback**  **3 times/weeks for 5 weeks**  (15 sessions) | 5 weeks | VAS  SF-36  ODI  RMDQ (follow up 5 weeks) | 5 weeks. IG demonstrated significant improvement at RMDQ , ODI , VAS and at SF-36 respect CG  There was a statistically significant increase in the scores of the patients in both groups |
| Neweeksome et al (49) | 30 patients  age 21-72  IG: 15  median age: 38 (27 to 43.5)  Gender (M%): 46  CG: 15  median age: 37 (30.5 to 45)  Gender (M%): 73 | early stage (2 hours after surgery) | **IG: early passive mobilization + standard exercises and advice sheets**  10 passive assisted and active hip/knee flexion exercises. The patient was encouraged to repeat this exercise every 30 minutes.  Mobility out of bed 4-5 hours after surgery.  **CG: standard exercises and advice sheets**  Mobility out of bed 4-5 hours after surgery without passive assisted and active hip/knee flexion exercise. | 4 weeks  12 weeks | The time taken for the patient to become independently mobile and attain discharge criteria following surgery (based on physiotherapist’s opinion)  ODI  VAS  Return to work  Short Form McGill pain Questionnaire | 4 weeks and 12 weeks. IG demonstrated significant improvement at the time reduced time to independent mobility and at Return to work respect CG |
| Beneck et al. (50) | 98 patients age 18-60  IG: 51  mean age: 39,2 ± 10,2  Gender (M%): 58%  CG: 47  mean age: 41,4 ± 9,9  Gender (M%): 50% | late stage (4-6 week after surgery) | **IG: education + supervised exercise**  One hour face to face with the PT and an educational booklet  Exercise program 3 times/week for 12 weeks (strengthening and endurance exercises of the back and leg in both supine, quadrupedal and standing positions).  **CG: education**  One hour face to face with the PT and an educational booklet | 12 weeks | SF-36 | 12 weeks. IG demonstrated significant improvement at SF-36 role physical, SF-36 bodily pain and SF-36 physical function when compared across the three groups |
| Jentoft et al. (51) | 80 patients  age 18-60  IG: 40  Mean age: 40.2 ± 10.2  Gender (M%): 67,6%  CG: 40  mean age: 39.4 ± 10.3  Gender (M%): 57,6% | early stage (1 day after surgery) | **IG: information and education + supervised exercise**  Patients received the same postoperative mobilization and information as the CG but were additionally given two to six treatment sessions depending on the length of their hospital stay. Seven exercises were performed in one set of 8–10 repetitions, twice a day at the hospital.  **CG: information and education**  Postoperative mobilization and information | 6-8 weeks  52 weeks | NPRS (leg and back pain),  ODI,  TSK-13 (13–52),  FABQ. | 6-8 weeks. No significant differences between the two groups for the outcomes  52 weeks. IG demonstrated significant improvement at NPRS (leg pain), and at ODI respect CG |
| Zhao et al. (52) | 69 patients  IG: 35  mean age: 52.3 ± 7.6  Gender (M%): 54,3%  CG: 34  mean age: 52.5 ± 8.9  Gender (M%): 58,8% | early stage (immediately after surgery) | **IG: deaquation and nerve nutrition medication**, (6 weeks in bed) + acupuncture (30 min, once/day, 15 days)  **CG: deaquation and nerve nutrition medication**, (6 weeks in bed) | 12 weeks  24 weeks  52 weeks | JOA  Functional recovery rates (postoperative score - preoperative)/15 preoperative score x 100% | 12 weeks. IG demonstrated significant improvement at JOA and Functional recovery rates respect CG  24 weeks. IG demonstrated significant improvement at JOA and at functional recovery rates respect CG  52 weeks. IG demonstrated significant improvement at Functional recovery rates respect CG |
| Ostelo et al. (53) | 105  age 18-65  IG: 52  Mean age: 42,8 ± 8,8  Gender (M%): 50%  CG: 53  mean age: 43,7 ± 8,8  Gender (M%): 64,2% | early stage | **IG: Behavioral Graded Activity (BGA)**  Individually tailored exercise program of increasing intensity, behavioral graded activity (operant therapy) using graded activity and positive reinforcement, time‐contingency management. Patients had to practice at home. 30 min/session over a three‐month period (max 18 sessions)  **CG: usual care**  The whole spectrum of techniques used by physiotherapists were included, but cognitive behavioral treatment, acupuncture, osteopathic techniques and all kinds of other “alternative” techniques were excluded. 30 min/session over a three‐month period (max 18 sessions) | 12 weeks | Global perceived effect (GPE) with the 7-point scale  RMDQ  VAS (severity of main complaint and severity of back pain)  SF-36 and resource costs | 12 weeks. CG demonstrated borderline significant improvement for GPE respect IG  No significant differences between the two groups for the outcomes measured by RMDQ, VAS and SF-36 |
| Erdogan et al. (54) | 62 patients  IG: 31  Mean age: 39,97±10,89  Gender (M%): 54.8%  CG: 31  mean age: 40,58 ±11,45  Gender (M%): 58.1% | early stage | **IG: education and information with computer-assisted training**  **CG: education and information with booklet** | 2 weeks  4 weeks  12 weeks | ODI  State-Trait Anxiety Inventory (STAI) | 15 days. IG demonstrated significant improvement at ODI and STAI respect CG  4 weeks. IG demonstrated significant improvement at ODI and at STAI respect CG  12 weeks. IG demonstrated significant improvement at ODI and at STAI respect CG |
| Ozkara et al. (55) | 30 patients  age 18-60  IG: 15  Mean age: 48.53 ±11.95  Gender (M%): 40%  CG: 15  mean age: 44.13 ± 8.88  Gender (M%): 46.7% | early stage (1 day after surgery) | **IG: information and education + unsupervised exercise**  Information and education given by the surgeon and the physical therapist and a home-based exercise program (2 series on days - 3 days /week for 12 weeks)  **CG: information and education**  Information and education given by the surgeon and the physical therapist. | 6 weeks  12 weeks | ODI  VAS  Beck Depression Inventory Scale  SF-36  Return to work | 6 weeks. No significant differences between the two groups were found regarding all outcomes  12 weeks. IG demonstrated significant improvement for VAS, ODI, PF and at SF-36 respect CG |
| Manniche et al.(56) | 62 patients  age 18-74  G: 31  Median age: 51  Gender (M%): 58,06%  CG: 31  Median age: 49  Gender (M%): 38,7% | deferred phase (14-60 month after surgery) | **IG: supervised exercise with hyperextension**  Intensive dynamic exercise with hyperextension, start session with hot pack (optional) (20 minutes), followed by trunk lifting, leg lifting, abdominal exercise.  Two sessions a week (one session: 60 to 90 minutes), total of 24 sessions in three months.  **CG: supervised exercise without hyperextension**  Exactly the same procedure, but in the 1st and 2nd exercise, the movement range of the back and hip is only from 90 degrees flexion to 0 degrees. No hyperextension allowed. | 12 weeks  48 weeks | LBPRS | 12-48 weeks. No significant differences between the two groups were found regarding all outcomes |
| Kim et al. (57) | 21 patients  age 25-69  IG: 14  Mean age: 45,7 ±12,4  Gender (M%): 36%  CG: 7  mean age: 54,9 ±6,7  Gender (M%): 71% | early stage (2-3 weeks after surgery) | **IG: manual therapy**  Osteopathic manipulative treatment. The protocol included manipulative and non-manipulative techniques without include high-velocity, low-amplitude thrust (HVLAT) manipulation of the lumbar segments where the surgery was performed. Eight individualized sessions, 30 min twice a week.  **CG: unsupervised exercise**  Home exercise program with booklet and verbal instruction. The active home exercise was recommended to perform twice a week for four weeks, each for half an hour | 104 weeks | VAS (0 -100) (leg and back pain)  RMDQ (0-24)  SF-36 | 104 weeks. IG demonstrated significant improvement for the disability item of RMDQ respect CG  No significant differences between the two groups were found regarding VAS (leg and back pain) |
| Abdi et all. (58) | 87 patients  IG: 28  mean age: 44.20  Gender (M%): 34.1%  CG: 29  mean age: 44.95  Gender (M%): 31.8% | Late phase 8at the end of the 6^th^ week after surgery) | The participants performed the exercises at home (8 weeks, 3 times a week for 45 min, on Sunday, Tuesday, and Thursday) without supervision.  **IG: unsupervised exercise (flexion-based Williams group )**  Participants in this group received seven exercises including the following: pelvic tilt, single knee to chest, double knee to chest, partial sit-up, hamstring stretch, hip flexor stretch, and squat.  **CG: unsupervised exercise (extension-based McKenzie group)**  These exercises include lying prone, prone on elbows, standing extension, lying flexion, sitting flexion, and standing flexion. | 14 weeks | VAS  ODI  mBST  TFET    Return to work | 14 weeks. All the groups significantly improved ODI and VAS, with a statistically significant difference in favour of the IG.  IG demonstrated significant improvement in trunk flexion endurance compared to CG.  CG demonstrated significant improvement in trunk extension endurance compared to IG.  No significant differences  between the two groups were found  regarding return to work. |
| Wang et all. (59) | 129 patients  IG: 63  mean age: 38,7 ± 7,6  Gender (M%): 54%  CG: 66  mean age: 35,2 ± 8,1  Gender (M%): 57,6% | Early stage  EG: the average period of follow-up  was 17.2 ± 4.2 (14–31) months.  CG: the average period of follow-up  18.8 ± 4.1 (14–33) months. | **IG: Supervised exercise**  A function exercise guiding group was assigned to manage  the functional exercises, which began at the time of admission and lasted until discharge.  **CG: Unsupervised exercise (usual care)**  For the control group, routine exercise program was applied  and the exercise began the nextday after operation. | 3^rd^ d  4 weeks  12 weeks  24 weeks  48 weeks | VAS  ODI | 3 day and 4 weeks. IG demonstrated significant improvement in VAS and ODI.  12 weeks, 24 weeks and 48 weeks. No significant differences were found  between the two groups regarding VAS.  IG demonstrated significant improvement in ODI compared to IG only at 12 weeks. No significant differences were found at 24 and 48 weeks. |

LEGEND

| ***Intervention*** |  |
| --- | --- |
| Advice | The reviewers decided to use the term advice to collect interventions like guidebook, booklet or an electronic file with advice about exercises and movements to do or not to do. |
| Cognitive Behavioral Treatment (CBT) | Pain Education, active coping strategies, Positive Reinforcement, Behavioral Graded Activity (BGA), Graded Exposure |
| Education | Patient education regarding the management of their postoperative clinical condition, including advice on ergonomics, also with specific booklet |
| Manual Therapy | Manual techniques, joint mobilization, myofascial techniques |
| Supervised exercise | Any form of exercise, both individually and in groups, carried out in the presence of a physiotherapist. Muscle strengthening exercises (isometric, concentric and eccentric activation), motor control exercises, mobility exercises, stretching, aerobic exercise have been included in this category. |
| Unsupervised exercise | Any form of exercise, as long as it is performed at home, be it illustrated in a booklet or previously explained by the physiotherapist before starting the exercise program at home. |
| Usual care | Procedure used within the ward, including instructions provided by the surgeon at the time of discharge and any physiotherapy treatments if required by the ward. |
| ***Outcome*** |  |
| CSQ | Coping Strategies Questionnaire |
| EuroQol-5D | European quality of life-5 dimensions |
| FABQ | Fear Avoidance Beliefs Questionnaire |
| JOA | Japanese Orthopaedic Association (JOA) score |
| LBPRS | Low Back Pain Rating Scale |
| mBTS | Modified Biering-Sorensen test |
| NPRS | Numeric Pain Rating Scale |
| ODI | Oswestry Disability Index |
| RMDQ | Roland & Morris Disability Questionnaire |
| SF-12 | Short Form Health Survey 12 |
| SF-36 | Short Form Health Survey 36 |
| TFET | Trunk Flexion Endurance Test |
| TSK | Tampa Scale of Kinesiophobia |
| VAS | Visual Analog Scale |
