# Supplementary material for: Rehabilitation after lumbar spine surgery in adults: a systematic review with meta-analysis
Source: Arch Physiother. 2023 Oct 16;13:21. doi: 10.1186/s40945-023-00175-4 (PMC10578022; doi:10.1186/s40945-023-00175-4)
Supplement: Supplementary file 3 — Additional file 3. [file 40945_2023_175_MOESM3_ESM.docx]

Appendix 3 - Qualitative analysis of the studies on lumbar disc herniation

*Comparison: supervised exercises vs unsupervised exercises: early stage*

One RCT (N=92) with high risk of bias (22) found improvements induced by supervised exercises in the quality of life at 6 months, compared to unsupervised exercises (p<0.05). One low risk-of-bias RCT (N=59) (20) instead showed at 12 months significant improvements in favor of the group performing home exercises on quality of life (p= 0.03) and pain (p= 0.040), nevertheless no differences in disability at 3-months follow-up. On the other hand, Lu et al. (19) suggested that disability was lower in experimental group than in the control one, both at 6 weeks and 3 months follow-ups.

*Comparison: supervised exercises vs no treatment: early stage*

Three studies (34, 35, 36) investigated the effectiveness of a supervised exercises program versus no treatment. Two studies (34, 35) were conducted on the same sample of participants and both studies consisted of 3 groups (i.e. physiotherapy, sham, no treatment). There were no common outcomes between these studies. Two studies had low risk of bias (34,35) while the study of Ju et al. (36) had high risk of bias.

Erdogmus et al. (34) (N= 120) found that the Low Back Pain Rating Score of physiotherapy-treated patients was significantly lower than in untreated patients (p=0.005), at short-term (12 weeks). There was no statistically significant difference between the physiotherapy-treated and the sham-treated groups, nor between the sham-treated and “no treatment” groups.

Ebenbichler et al. (35) (N=120) found that the group undergoing comprehensive physiotherapy had significantly better functional outcomes, as rated on the Low Back Pain Rating Score, than the untreated group (p=0.03) at long-term (12 years after surgery). However, these three-arm studies pointed out that there was no significant difference between intervention and sham groups.

Ju et al. (36) (N= 14) found pain improvements in favor of the group undergoing supervised exercises (p<0.05) at early stage. Nevertheless, the small sample of this study (7 patients per group) may influence interpretation of results.

As a summary of these results, there were improvements in the groups undergoing supervised physiotherapy compared to the "no treatment" group, especially for pain reduction.

*Comparison: exercises (supervised and unsupervised) vs manual therapy: early stage*

Two pilot studies on very small samples (37,38) analyzed the effectiveness of supervised (37) and unsupervised (38) exercises programs compared to a manual therapy program. Both studies found improvements on pain and disability at short-term (4 weeks after intervention, 7-8 weeks after surgery) in favor of the manual therapy group.

Kim et al. (37) found pain improvements in the manual therapy group with 53% reduction from baseline compared to the exercises group, which had 17% reduction. Residual LBP also decreased in both interventions, with 37% reduction in the manual therapy group and 10% reduction in the exercises group.

Kim et al. (38) found improvements in early post-operative LBP in both groups with 47% reduction in the manual therapy group and 43% reduction in control care group. However, early post-surgical leg pain was more decreased by manual therapy group (55% reduction), than active control care (9% reduction).

In both studies (37,38) post-surgical physical disability was more improved in the manual therapy group. The risk of bias of these studies was low (37) to moderate (38).

*Comparison: supervised exercises + education versus education: late stage*

Two RCTs (30, 31) studied the effects on disability of supervised exercises and education compared to education alone. In the study of Kulig et al. (30) with high risk of bias (N=98), a third group (usual physical therapy group) was created post-hoc due to the high number of deviations from the original assigned group of intervention. Comparisons showed most improvement in the exercises plus education group compared with both the education group (p<0.016) and the usual physical therapy group (p<0.003).

Differently, McGregor et al. (31) in their large study with moderate risk of bias (N=338) did not find any significant difference between the two groups.

*Other results*

Some RCTs were also found without any possibility of comparison with other studies due to the different interventions delivered.

A study with low-risk of bias (Hebert, 2013) (21) (N=61) compared a trunk-specific exercises program to a general exercises program, both performed under supervision. No difference between the groups in clinical and muscle function outcomes was reported.

The study with low risk of bias by Hakkinen et al. (39) (N=126) compared an intervention group who performed home strengthening exercises combined with stretching with a control group performing only stretching. At the 12-month follow-up, no statistically significant changes were found in physical function, pain or disability between the groups.

In the work with high risk of bias by Manniche et al. (40) (N=62), the intervention group performed high intensity exercises with lumbar hyperextension while control group without

hyperextension; the added use of hyperextension exercises did not induce any further benefit.

The work with low risk of bias by Zoia et al. (41) (N=54) showed that corset adoption did not improve short-term and mid-term outcomes (pain and disability) in patients after one level lumbar discectomy.

The study with high risk of bias by Kara et al. (42) (N=54) investigated the immediate effectiveness of the Transcutaneous Electrical Nerve Stimulation **(**TENS) added to patient-controlled analgesia (PCA) compared to only PCA and showed that TENS was an effective application in reducing activity-related pain, side effects related to analgesic consumption, and medication use (N= 54).

Bono et al. (43) (N=112) investigated the effects on having a restriction of movement in the two post-surgical months compared to having them for six months. The results of this RCT with high risk of bias suggested equivalent clinical outcomes irrespective of the length of post-operative restriction.

Aldemir et al. (44) (N= 80) in a low risk of bias study compared a group of patients who had indications to walk using a pedometer to a control group receiving education sessions. The pedometer-supported walking and telemonitoring intervention in the early post-operative period increased the physical activity levels of the patients, decreased their pain and disability and increased their quality of life.

Rothhaupt et al. (45) (N= 32) compared in a high risk of bias study a group of patients taking part to hippotherapy classes compared to a control group who performed spa therapy. The results showed that patients who did hippotherapy had faster results on the ability to work rather than the control group.

Reyes et al. (46) (N=24) studied in a high risk of bias trial the comparison between supervised exercises alone and supervised exercises with the addition of neural mobilizations. A standard rehabilitation, alone or in combination with neural mobilization techniques, was similarly effective in reducing pain and disability, and improving health-related quality of life.

Ostelo et al. (47) (N=105) compared cognitive behavioral therapy interventions with educational sessions. This study with moderate risk of bias concluded that there were no differences between the two treatments on clinical outcomes, but behavioral-graded activity was associated with higher costs.

Kacar et al. (48) (N=20) investigated the effect of a pressure biofeedback device employed during supervised exercises compared with its non-use. This study with high risk of bias showed that patients who exercised with a biofeedback returned to everyday life with higher functionality.

Newsome et al. (49) (N=30) studied in a high risk of bias trial the effects of passive mobilizations some hours after surgery before getting out of bed, versus alone standing mobilization of the patient. The immediate treatment enabled patients to recover independent mobility and return to work more rapidly.
Beneck et al. (50) (N=98) allocated participants in two groups: one received exercise combined with education and the other education alone. An intensive, progressive exercise program combined with education increased quality of life in these patients.

Jenoft et al. (51) (N=80) compared one group that received information with one group that performed exercise in combination with information. The authors concluded that exercise in combination with information reduced leg pain and improved function.

Zhao et al. (52) (N=69) investigated the clinical effects of acupuncture after surgical operation. A group of patients was treated by acupuncture and conventional rehabilitation therapy and the other one only by rehabilitation therapy. The authors showed that acupuncture could improve the functional recovery for patients operated for disk prolapse.

Ostelo et al. (53) (N=105) analyzed the cost-effectiveness of a behavioral-graded activity program compared to usual care. No differences were found between the two treatments.

Erdogan et al. (54) (N=62) tried to identify the effects of computer assisted training schemes versus education through a booklet and concluded that this method was efficient as an educational tool for patients undergoing lumbar disc herniation.

Ozkara et al. (55) (N=30) compared patients that followed an unsupervised exercise program to a control group that did not perform any kind of exercise. There was an improvement in pain, disability and spinal function in the exercise group.

Manniche et al. (56) (N=62) examined the effects of a high intensity exercise program compared to a mild one. In the first case, participants had better results in terms of work capacity improvements.

Kim et al. (57) (N=21) assessed the feasibility of manual therapy to improve late post-operative outcomes and the results showed that post-operative physical disability improved more in patients undergoing rehabilitation, than in those receiving control care.

Abdi et al. (58) (N=87) examined the effects of two different unsupervised exercises, one more characterized by flexion-based and one by extension-based exercises, after 6 weeks of lumbar discectomy. The results after 14 weeks showed a VAS score reduction and ODI improvement more in the extension-based exercises, than in the flexion-based exercised group. There was no significant difference between groups in terms of return to work.

In the end, Wang et al. (59) (N= 127) assessed VAS and ODI in two different groups after lumbar discetomy: the experimental group underwent supervised exercises before and after the surgery, instead the control group underwent unsupervised exercises like routine program. The VAS score was significantly lower in the pre-operative supervised exercises, than the unsupervised exercises group at 3 days and 1 month after surgery; also in this article was found that ODI at 1th and 3th month after surgery was better in the experimental, than the control group.
